# Social Status Affects the Degree of Sex Difference in the Songbird Brain

**DOI:** 10.1371/journal.pone.0020723

**Published:** 2011-06-08

**Authors:** Cornelia Voigt, Manfred Gahr

**Affiliations:** Department of Behavioural Neurobiology, Max Planck Institute for Ornithology, Seewiesen, Germany; Pennsylvania State University, United States of America

## Abstract

It is thought that neural sex differences are functionally related to sex differences in the behaviour of vertebrates. A prominent example is the song control system of songbirds. Inter-specific comparisons have led to the hypothesis that sex differences in song nuclei size correlate with sex differences in song behaviour. However, only few species with similar song behaviour in both sexes have been investigated and not all data fit the hypothesis. We investigated the proposed structure – function relationship in a cooperatively breeding and duetting songbird, the white-browed sparrow weaver (*Plocepasser mahali*). This species lives in groups of 2–10 individuals, with a dominant breeding pair and male and female subordinates. While all male and female group members sing duet and chorus song, a male, once it has reached the dominant position in the group, sings an additional type of song that comprises a distinct and large syllable repertoire. Here we show for both types of male – female comparisons a male-biased sex difference in neuroanatomy of areas of the song production pathway (HVC and RA) that does not correlate with the observed polymorphism in song behaviour. In contrast, in situ hybridisation of mRNA of selected genes expressed in the song nucleus HVC reveals a gene expression pattern that is either similar between sexes in female – subordinate male comparisons or female-biased in female – dominant male comparisons. Thus, the polymorphic gene expression pattern would fit the sex- and status-related song behaviour. However, this implies that once a male has become dominant it produces the duetting song with a different neural phenotype than subordinate males.

## Introduction

Sex differences in brain structure and behaviour are widespread among vertebrates. In mammals, including humans, numerous studies have accumulated evidence for sex differences in brain anatomy, neurochemistry and activity. However, in most cases their functional significance is still unknown [1]–[3]. In contrast to most mammalian neural circuits with sex differences, the song control system of songbirds is a functionally well-defined neural circuit that has become a widely-used model for the study of brain-behaviour relationships (for review, [4]). This network of forebrain areas, responsible for song learning and song production, was found to exhibit extreme sex differences in size and neuron number in some species [5]. For example, in zebra finches (*Taeniopygia guttata*), where only the male sings, the volumes of song control nuclei RA and HVC are about 5 times larger in males than in females, and the song nucleus area X is not even recognizable in females [5]. In contrast, in bay wrens (*Thryothorus nigricapillus*), a duetting species with similar song in males and females, RA, HVC and area X are only about 1.1–1.5 times larger in males compared to females [6]. These anatomical sex differences are thought to be functionally related to sex differences in vocal behaviour. Interspecific comparisons have reinforced the view that sex differences in song system size have co-evolved with sex differences in vocal behaviour and hence, those species with similar song in both sexes have similar-sized song control areas [7]–[9]. However, findings from two duetting songbird species with monomorphic singing, provide evidence that similar song performance in males and females does not necessitate similar neural gross morphology [10], [11].

Sexually dimorphic phenotypes result to a large extent from differential expression of genes that are present in both sexes because male and female genomes only differ by those genes located on sex-specific chromosomes. Such sex-biased expression was documented for thousands of genes in somatic tissue of mice [12]. In birds, microarray studies have obtained similar results, although to a much smaller extent in the embryonic chicken brain [13] and recently in the songbird brain at different developmental stages [14], [15]. How these expression patterns might change throughout life in response to environmental and social cues is yet largely unknown. In the current study, besides the analysis of overall song system neuroanatomy, we focused on possible sex differences in expression levels of selected genes in the major song nucleus HVC. The song control system is a steroid-sensitive neural circuit that comprises distinct pathways for song learning and song production, and shows high morphological and neurochemical plasticity both during development and in adult life (for review, [16]). Androgens and estrogens are known to play a major role in those processes and are important for the regulation of synaptic plasticity [17]. Therefore, we investigated steroid hormone receptors and synapse-associated proteins, whose expression patterns within the song system had been described previously [18], [19]. All but one of the selected genes (syntaxin 1B) are known to be autosomal in the chicken. Thus, assuming a similar location in our species, a male-bias in gene expression due to the limited gene dosage compensation in birds can be excluded [20]. The chromosomal location of syntaxin 1B is yet unknown.

Our study species, the white-browed sparrow weaver (*Plocepasser mahali*), is a cooperatively breeding and duetting songbird that exhibits a pronounced dominance hierarchy and a polymorphism in song behaviour. While all males and females sing duet and chorus songs, dominant males differ from subordinate males in singing an additional type of song, which comprises a distinct and large syllable repertoire [21]. This feature offers the opportunity to study neural sex differences among different groups of individuals within the same species, i.e. those that differ in song behaviour (dominant males and females) and those that have the same song output (subordinate males and females). White-browed sparrow weavers are widespread throughout Africa and are common residents in southern Zimbabwe [22]. They live in groups of 2–10 individuals in year-round territories with a single dominant breeding pair and male and female subordinates [23], [24]. Their song behaviour has been described in detail previously [21].

Here we asked whether the polymorphic song behaviour is reflected in the overall size of the song system and/or its gene expression patterns. According to the proposed structure – function hypothesis there should be anatomical sex differences among individuals that differ in song behaviour, but not among those that have a similar song pattern. Therefore, we compared, among dominant males, dominant females and subordinate males gross-morphology of song nuclei HVC, RA and area X. HVC and RA are part of the descending motor control pathway of the song control circuit and involved in the generation of song motor patterns, while area X is part of the anterior forebrain pathway and likely to play a role in song sensorimotor learning and context dependency of singing [25], [26]. By using *in situ* hybridization, we measured the mRNA expression levels of the androgen and estrogen receptors and the synapse-associated proteins SNAP-25, synaptoporin and syntaxin 1B in the major song nucleus HVC.

## Methods

### Ethics statement

All research was approved by the Research Council of Zimbabwe (Executive director, Cabinet office, P.O.Box CY294, Causeway, Harare, Zimbabwe) and conducted in accordance with the permit obtained from this institution (Permit No. 02240).

### Animals

Birds (dominant males, N = 8; subordinate males, N = 8; dominant females, N = 8) were sampled in southwestern Zimbabwe (20°08′S–20°14′S; 28°56′–29°01′E) during the rainy season (January to March in 2000 and 2001) and derived from groups of individually known, colour-ringed birds. The breeding season of white-browed sparrow weavers coincides with the rainy season, which lasts from November until the end of March. Birds were captured shortly after dusk (7–9 pm) inside their roosting nests. The sex was determined according to bill colour [27] and verified upon sacrifice of the bird. Each sparrow weaver group consists of a single dominant pair and several male and female subordinates. We identified the dominant individuals by breeding activities, their frequent duetting and their chasing of other group members. The dominant male was usually the last bird to enter the roosting nest in the evening and it was the only group member that performed the solo song at dawn [21]. We verified this by observing each group thrice at dusk and at dawn. Additional male group members, which were fully mature but did not show the behaviour of the dominant male, were considered subordinate. Birds captured for neuroanatomical analyses were kept overnight singly in cages until sacrifice on the following morning. None of the birds showed singing behaviour during this time.

### Song recording and analysis

Vocalisations were recorded with a Sony TCD-5M cassette recorder (Sony Corp., Tokyo, Japan) equipped with a Sennheiser ME-88 directional microphone (Sennheiser electronic, Wedemark, Germany). Male solo song was recorded in the morning between 5:00 and 5:45. In previous observations, we determined the approximate starting time, which was generally coincident with or slightly before first light. Solo song was only produced once a day at dawn. Duet songs were performed throughout the day, and recordings were made between 5:45 and 19:00. Details on sonographic analysis are described elsewhere [21].

### Neuroanatomical analysis

Birds were killed with an overdose of chloroform, and perfused transcardially with 0.9% saline, followed by 4% phosphate buffered formaldehyde. After fixation, brains were freeze-protected with 15%, followed by 30% phosphate-buffered sucrose at 4°C, then cut into 30 µm parasagittal sections and mounted onto Superfrost-Plus slides (Roth). One series of sections was used for Nissl staining; the others were processed with *in situ* hybridization for androgen receptor (AR) estrogen receptor α (ERα; [18], synaptosomal-associated protein (SNAP-25; GenBank: AY531112), synaptoporin (SPO; GenBank: AY531113) and syntaxin 1B (STX1B; GenBank: GQ374456). The mRNA expression on brain sections was detected with zebra finch antisense RNA probes labelled with ^35^S-CTP and followed a standard protocol with modifications [28]. The cloning of the partial zebra finch AR, ERα, SNAP-25 and SPO cDNAs were done in our laboratory and have been described previously [18], [19]. Based on sequence information available, PCR was used to amplify a fragment of the *STX1B* gene from zebra finch. The mRNA was prepared from brain tissue by using the RNeasy Mini Kit (Qiagen GmbH, Hilden, Germany). The synthesis of first-strand cDNA was done with SUPERSCRIPT II Reverse Transcriptase (Invitrogen, Karlsruhe, Germany) and oligo (dT)-primer. The resulting RNA-DNA hybrids were subsequently used in PCR to generate pieces of the appropriate gene. For *STX1B* the forward primer was 5′-TTYGAGCARGTNGARGARAT-3′ and the reverse primer was 5′-GCCATRTCCAYRAACATRTC-3′. PCR was carried out for 40 cycles by using the following parameters: 94°C for 1 minute, 52°C for 45 seconds, 72°C for 1 minute. Amplified fragments were purified, blunt-ended and cloned into the Sma I site of the plasmid vector pGEM7ZF (Promega, Mannheim, Germany). Resultant clones were sequenced to verify the authenticity and fidelity of the amplification. The cloned *STX1B* sequence is 560 bp in length and is 87% identical to its human counterpart (Genbank AY028792).

### Morphometric analysis

Slides were analyzed under brightfield and darkfield illumination using a Leitz Aristoplan microscope (Leitz Wetzlar, Germany) and the brain regions HVC and RA were video-digitized using an image analysis system (MetaMorph 4.6, Visitron Systems, Germany). Volumes were calculated from every fourth section as the sum of the area sizes multiplied by 120 µm (section interval × section thickness). Telencephalon volume was estimated by sampling every eighth section throughout the extent of the brain hemisphere. Total volume was the sum of the measurements from the right and the left hemisphere. Males are larger than females in terms of overall size, such as body weight and wing length [29]. To correct for variation in body and brain size we used measurements of HVC, RA and area X volume relative to telencephalon size (song nucleus volume/telencephalon volume). Cell density in HVC and RA was estimated from Nissl-stained sections under high magnification with help of the image analysis system Metamorph 4.6. In each animal at the lateral, central and medial level of the nucleus (see below), a counting frame of 10,000 µm^2^ for HVC and 62,000 µm^2^ for RA was analysed using the digitised images, and the average of these counts was calculated. We sampled a minimum of 160 cells in each nucleus per bird. We counted all profiles that contained one or two nucleoli throughout the entire depth (30 µm) of the section that fell within the boundaries of the counting frame. Density measurements are presented as 10^4^ cells/mm^3^. The total number of cells in each nucleus was derived from multiplying cell density by the volume of the nucleus. The mRNA expression in HVC was measured at the lateral, central and medial level of the nucleus. These levels were estimated according to the Nissl-defined boundaries of HVC. At each level four areas (13,100 µm^2^ each) across HVC were analyzed (in total 52,400 µm^2^). To quantify the level of mRNA expression in an area, the image was converted to a greyscale image. A threshold level was then adjusted to separate the silver grains from the background. The above-threshold fraction of the area was calculated by a built-in function of the software. The mean of these measurements was named mRNA expression level. To correct for different amounts of background labelling, we measured the area covered by silver grains in a region of the same section lacking specific labelling. Correction was done by subtracting the value for background labelling from the value of HVC. In our species, ER mRNA is only expressed in medial HVC and therefore measurements were only derived from that region. For dominant males and females, data for this gene could only be obtained from respectively 6 individuals. For syntaxin 1B data were only available from 7 females. A subset of the male data was reported in a previous study [28].

### Statistical analysis

Statistical analyses were carried out using Systat v. 12.0 (Systat Software Inc.). Data are presented as mean ± s.e.m. Morphological data were compared using General Linear Models with group (dominant males, subordinate males, dominant females) as factor. Posthoc comparisons were performed with Tukey's HSD test. Correlations were calculated with Pearson correlation. Because males and females differed significantly in HVC cell density, the gene expression levels in this nucleus were analysed using ANCOVA, with group as factor, and cell density as covariate. All tests were two-tailed, and the significance level was set at *p*<0.05.

## Results

### Song behaviour

**Figure 1:**
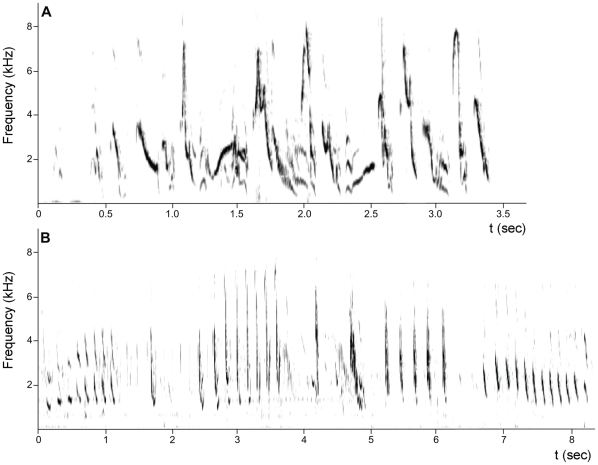
White-browed sparrow weavers possess an extraordinary vocal communication system with two completely different types of song. Sonagrams from field recordings showing a duet song (A, similar in structure to chorus song) and a sequence from solo song (B) of white-browed sparrow weavers. While all group members engage in duet and chorus singing, solo song is only produced by the dominant male of the group. Note, that time scales are different.

Songs of white-browed sparrow weavers can be divided into solo songs, duets, and chorus songs. The structure of chorus songs resembles that of duets except that more than two individuals participate. Choruses were frequently heard during aggressive encounters with neighbouring groups. Song characteristics, including repertoire size and song length, have been described in detail in a previous study [21]. The main results for those birds that were used in the present study are summarized below. All group members engage in duet and chorus singing and have similar-sized repertoires (mean: 51.9±2.1syllable types, Fig. 1A). Mean duet length is 2.84±0.11 s and on average 4.98±0.10 syllables per second are sung. Solo singing, in contrast, lasts on average 740.0±114.9 s and involves an additional large syllable repertoire (mean: 68.9±6.1 syllable types, Fig. 1B). Syllable overlap between both types of song averages at 2.1±0.6%. Solo song is exclusively performed by the dominant male of the group. Thus, total repertoire size of dominant males (comprising duet and solo song repertoire) consists of 128.3±8.3 syllable. All males and females sang duet and chorus song. All dominant males, but none of the subordinate males, were observed singing solo song.

### Neural sex differences

**Figure 2:**
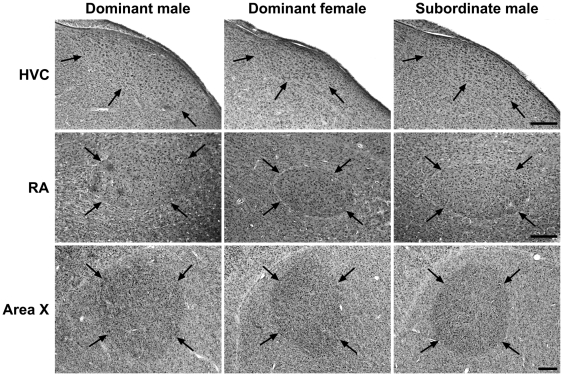
Volumes of song control nuclei HVC and RA but not area X are larger in males than in females. Photomicrographs of Nissl-stained HVC, RA and area X of dominant males, dominant females and subordinate males are shown (parasagittal sections; arrows indicate boundaries of the nuclei; scale bars = 300 µm).

**Figure 3:**
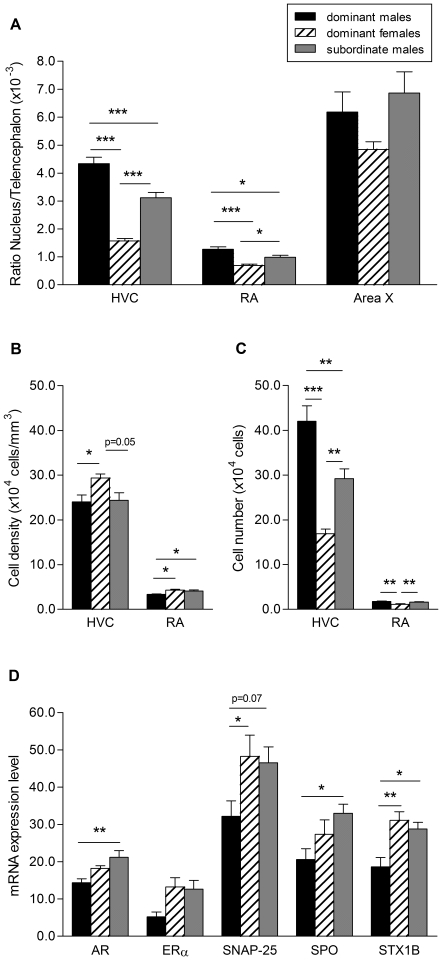
Sex differences in gross-morphological and cytochemical features of the vocal control system between dominant males and females and subordinate males. Ratio nucleus volume/telencephalon volume (A), cell density (B) and cell number (C) of song control nuclei HVC and RA. D: mRNA expression levels (fractional area covered by silver grains) of AR, ERα, SNAP-25, SPO and STX1B in HVC; (***p<0.001, **p<0.01, *p<0.05).

Gross-morphological analysis of the brain revealed significant differences between groups in the Nissl-defined volumes of HVC and RA but not area X (HVC: F_2,21_ = 60.61, p = 0.0001; RA: F_2,21_ = 17.58, p = 0.0001; Area X: F_2,21_ = 2.70, p = 0.091; Fig. 2, 3A, Table S1). Volumes of HVC and RA were respectively 2.7 and 1.8 times larger in dominant males than in dominant females (Tukey's HSD test, p<0.001 for both comparisons) and 2.0 and 1.5 times larger in subordinate males than in dominant females (HVC: p<0.001, RA: p<0.05). Intra-sexually, dominant males had 1.4 and 1.3 times larger volumes of HVC and RA than subordinates (HVC: p<0.001, RA: p<0.05). These gross anatomical sex differences in HVC and RA resulted from significant differences in both cell spacing and cell number (HVC, cell density: F_2,21_ = 4.52, p = 0.023; cell number: F_2,21_ = 26.30, p = 0.0001; RA, cell density: F_2,21_ = 6.06, p = 0.008, cell number: F_2,21_ = 10.27, p = 0.001; Fig. 3 B, C; Table S1). Cell numbers in HVC and RA were higher in both dominant and subordinate males compared to dominant females (p<0.01 for all comparisons). Cell densities in HVC and RA were higher in dominant females than in dominant males (p<0.05 for both comparisons) but not significantly different between dominant females and subordinate males (HVC: p = 0.05, RA: p = 0.822). Intra-sexually, cell numbers in HVC but not RA were higher in dominant than subordinate males (HVC: p<0.01, RA: p = 0.771). Cell density in HVC did not differ between males (p = 0.985) while cell density in RA was higher in subordinates than dominants (p<0.05). Solo song repertoire size of dominant males was neither significantly correlated with the volume of HVC (r_s_ = 0.27, p = 0.515) nor with RA (r_s_ = 0.55, p = 0.162).

**Figure 4:**
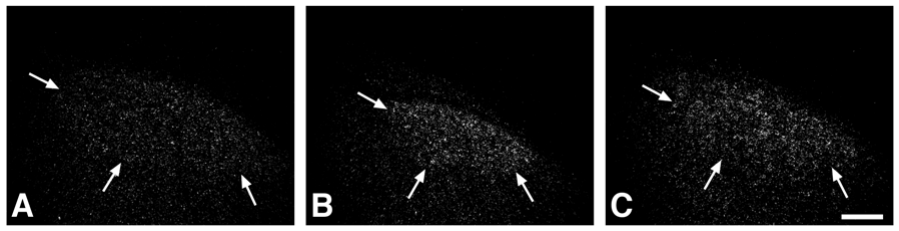
Expression of syntaxin 1B in HVC is higher in dominant females than in dominant males, but similar to subordinate males. Darkfield photomicrographs showing the expression of syntaxin 1B (STX1B) in song nucleus HVC of a dominant male (A), a dominant female (B) and a subordinate male (C). Arrows indicate the ventral border of HVC. Dorsal is to the top and caudal is to the right in these parasagittal sections, (scale bar = 300 µm).

The mRNA expression levels in HVC of four of the five genes studied revealed significant differences between groups (AR: F_2,20_ = 7.10, p = 0.005; ERα: F_2,16_ = 2.72, p = 0.096; SNAP-25: F_2,20_ = 5.77, p = 0.011; SPO: F_2,20_ = 4.86, p = 0.019; STX1B: F_2,19_ = 8.07, p = 0.003; Fig. 3 D, Table S1). Expression levels of two synaptic proteins, SNAP-25 and STX1B were higher in dominant females than in dominant males (SNAP-25: p<0.05, STX1B: p<0.01, Fig. 4) while the expression levels of the steroid hormone receptors and the synaptic protein SPO were similar between the sexes (p>0.09 for all tests). No sex differences were found in the expression levels of all five genes between dominant females and subordinate males (p>0.40 for all tests, Fig. 4) Intrasexually, subordinate males had higher expression levels of androgen receptor (p<0.01) and the synaptic proteins SPO and STX1B (p<0.05 for both tests) but not SNAP-25 (p = 0.07) compared to dominant males. There was no effect of cell density on gene expression levels (p>0.05).

## Discussion

We report that in a duetting songbird with a complex social system, the extent of sex differences within the song control system varies according to the males' dominance rank. While dominant male – subordinate male comparisons of the descending song control pathway correlate with status-related differences in singing [30], the present male – female comparisons do not fit the polymorphism found in song behaviour of white-browed sparrow weavers. Instead, the gross morphological differences of the descending song control areas were always male-biased whereas the gene expression patterns in HVC were either similar between sexes or female-biased. In other words, females appear to have the same neuronal phenotype as subordinate males but their nuclei contain fewer of these neurons, while dominant males have even more neurons and of a different type than subordinate males. Further, the size of forebrain song nucleus area X, not directly involved in patterning of adult songs but in sensorimotor learning, did neither correlate with sex nor status.

Dominant male white-browed sparrow weavers sing duet and chorus songs together with other group members; additionally they produce a solo song comprising a large syllable repertoire. Thus, compared to dominant females, their total song repertoire is about 2.5 times larger. In relation, volumes of HVC and RA are respectively 2.7 and 1.8 times larger than in females. This finding fits the hypothesis that differences in song behaviour are correlated with differences in brain structure. However, our data do not support the relationship when considering the comparison of subordinate males and dominant females. According to their similar song output, similar-sized song control areas would have been expected. On the contrary, subordinate males have respectively 2.0 and 1.5 times larger volumes of HVC and RA than dominant females.

This discrepancy could reflect the fact that males probably have a much larger silent than overt repertoire, i.e. all males would go through an ontogenetic period of sensorimotor learning during which they acquire the solo song, but as adults, subordinates are suppressed from producing it. In relation, all males are potentially able to acquire a dominant position and to produce solo song while females do not. Thus, this scenario would fit the “brain space for a learned task” hypothesis posited by Nottebohm et al. [31]. This hypothesis is based on the assumption that the number of song types a bird can sing is a function of the number of neurons in the respective brain areas [7]. However, taking up the dominant position is associated with the production of a new type of song and an increase in the size of song areas in males only. This now leads to the mutually exclusive hypotheses, namely that for the acquisition of solo song either the sex difference (female – subordinate male) or the male intra-sex difference (subordinate – dominant) in HVC size and neuron numbers is a pre-condition. If the first hypothesis is correct, a possible ultimate explanation for the increase in HVC neuron numbers while becoming dominant might be found in the more complex auditory scene to which the dominant male needs to respond. Solo singing males often perform at the same time, and neighbours frequently overlap each other. Song overlapping and matched countersinging have been shown in many species to signal threat and aggression [32] and they could well play a role in territorial defence of neighbouring groups of white-browed sparrow weavers. The representation of auditory social memories in HVC has not been studied in detail. Nevertheless, different HVC relay neurons appear to respond to different song types in playback studies of the birds own songs in swamp sparrows [33]. This type of auditory representation might facilitate patterns of song use such as song matching with neighbouring conspecifics.

If the second hypothesis is correct, sex differences in HVC size and neuron numbers would be rendered meaningless for song sensorimotor learning and production. Indeed, since adult females are able to sing solo song following testosterone treatment, (Voigt, unpublished observations) we think that the larger song areas and cell numbers of subordinate males are not required for solo song development. Such a correlation, initially proposed by Nottebohm et al. [31], [34], also failed to be confirmed in several other studies on different species [10], [11], [35]–[37]. Likewise, in species where song rate was found to be similar between sexes or even female-biased, HVC size was always male-biased [38]–[40].

In contrast to HVC and RA, the volume of area X in white-browed sparrow weavers did neither differ between sexes nor between dominant and subordinate males. Heterogeneity in the degree of sexual dimorphism has been reported before. In streak-backed orioles (*Icterus pustulatus*), for example, the size of area X and HVC is male-biased despite similar song complexity and female biased song rates [40]. In Bengalese finches (*Lonchura striata*) and brown-headed cowbirds (*Molothrus ater*), where females never sing, similar-sized volumes of LMAN of males and females have been attributed to female song discrimination ability [41], [42]. In white-browed sparrow weavers, the lack of sex- and status-related differences in area X volume might reflect their similar capacity for sensorimotor learning, which would be supported by our behavioural observations that all males and females, following testosterone treatment, are able to develop solo songs. However, this conclusion requires caution in the light of the problematic correlation between song phenotypes and neural phenotypes of HVC and RA in white-browed sparrow weavers (see above).

How do molecular features of HVC neurons correlate with song behaviour? These data indicate similar gene expression in neurons of subordinate males and females and lower expression in those of dominant males. Although our analysis concerns only a few genes, the results are likely to be meaningful (assuming that they will hold at the level of the protein). The synapse-related genes SNAP-25 and syntaxin 1, constituting components of the SNARE complex, are essential in the process of Ca^2+^-triggered exocytosis in neurons and neuroendocrine cells, confirmed by the study of gene-targeted mouse mutants [43], [44]. Thus, singing solo song would coincide with a HVC-wide lowered expression of synapse-related proteins. This observation, however, questions the relationship between gene-expression and song behaviour in subordinate males and females. Alternatively, if such a relationship would be meaningful for duet and chorus singing, then the neural representation of the duet/chorus song must change once a male becomes dominant; i.e. the neural mechanisms to produce the same type of song (duet/chorus) would differ even within the same sex. Clearly, this conclusion should be substantiated by the analysis of the entire HVC-transcriptom of dominant and subordinate males and females.

## Supporting Information

Table S1 **Measurements of neural properties of male and female white-browed sparrow weavers.**
(DOC)

## References

[1] Gahr M, Short RV, Balaban E (1994). Brain structure: causes and consequences of brain sex.. The Differences between the Sexes.

[2] Cahill L (2006). Why sex matters for neuroscience.. Nat Rev Neurosci.

[3] De Vries GJ, Södersten P (2009). Sex differences in the brain: The relation between structure and function.. Horm Behav.

[4] Nottebohm F (2005). The neural basis of birdsong.. PLoS Biol.

[5] Nottebohm F, Arnold AP (1976). Sexual dimorphism in vocal control areas of the songbird brain.. Science.

[6] Brenowitz EA, Arnold AP (1986). Interspecific comparisons of the size of neural song control regions and song complexity in duetting birds: evolutionary implications.. J Neurosci.

[7] Brenowitz EA (1997). Comparative approaches to the avian song system.. J Neurobiol.

[8] MacDougall-Shackleton SA, Ball GF (1999). Comparative studies of sex differences in the song control system of songbirds.. Trends Neurosci.

[9] Ball GF, Riters LV, MacDougall-Shackleton SA, Balthazart J, Zeigler HP, Marler P (2008). Sex differences in brain and behaviour and the neuroendocrine control of the motivation to sing.. Neuroscience of birdsong.

[10] Gahr M, Sonnenschein E, Wickler W (1998). Sex difference in the size of the neural song control regions in a dueting songbird with similar song repertoire size of males and females.. J Neurosci.

[11] Gahr M, Metzdorf R, Schmidl D, Wickler W (2008). Bi-directional sexual dimorphisms of the song control nucleus HVC in a songbird with unison song.. PLoS ONE.

[12] Yang X, Schadt EE, Wang S, Wang H, Arnold AP (2006). Tissue-specific expression and regulation of sexually dimorphic genes in mice.. Genome Res.

[13] Scholz B, Kultima K, Mattsson A, Axelsson J, Brunström B (2006). Sex-dependent gene expression in early brain development of chicken embryos.. BMC Neurosci.

[14] London SE, Dong S, Replogle K, Clayton DF (2009). Developmental shifts in gene expression in the auditory forebrain during the sensitive period for song learning.. Dev Neurobiol.

[15] Tomaszycki ML, Peabody C, Replogle K, Clayton DF, Tempelman RJ (2009). Sexual differentiation of the zebra finch song system: potential roles for sex chromosome genes.. BMC Neurosci.

[16] Brenowitz EA, Zeigler HP, Marler P (2008). Plasticity of the song control system in adult birds.. Neuroscience of birdsong.

[17] Gahr M (2007). Sexual differentiation of the vocal control system of birds.. Adv Genet.

[18] Gahr M, Metzdorf R (1997). Distribution and dynamics in the expression of androgen and estrogen receptors in vocal control systems of songbirds.. Brain Res Bull.

[19] Voigt C, Metzdorf R, Gahr M (2004). Differential expression pattern and steroid hormone sensitivity of SNAP-25 and synaptoporin mRNA in the telencephalic song control nucleus HVC of the zebra finch.. J Comp Neurol.

[20] Arnold AP, Itoh Y, Melamed E (2008). A Bird's-eye view of sex chromosome dosage compensation.. Ann Rev Genom Human Genet.

[21] Voigt C, Leitner S, Gahr M (2006). Repertoire and structure of duet and solo songs in cooperatively breeding white-browed sparrow weavers.. Behaviour.

[22] du Plessis MA, Hockey PAR, Dean WRJ, Ryan PG (2005). White-browed Sparrow-Weaver *Plocepasser mahali*.. Roberts birds of southern Africa (7^th^ edn).

[23] Collias NE, Collias EC (1978). Cooperative breeding behavior in the white-browed sparrow weaver.. Auk.

[24] Lewis DM (1982). Cooperative breeding in a population of white-browed sparrow weavers *Plocepasser mahali*.. Ibis.

[25] Hahnloser RH, Kozhevnikov AA, Fee MS (2002). An ultra-sparse code underlies the generation of neural sequences in a songbird.. Nature.

[26] Hessler NA, Doupe AJ (1999). Social context modulates singing-related neural activity in the songbird forebrain.. Nat Neurosci.

[27] Earle RA (1983). An attempt at sexing white-browed sparrow-weavers.. Safring News.

[28] Whitfield HJ, Brady LS, Smith MA, Mamalaki E, Fox RJ (1990). Optimization of cRNA probe in situ hybridization methodology for localization of glucocorticoid receptor mRNA in rat brain: a detailed protocol.. Cell Mol Neurobiol.

[29] Leitner S, Mundy P, Voigt C (2009). Morphometrics of white-browed sparrow-weavers *Plocepasser mahali* in south-western Zimbabwe.. Ostrich.

[30] Voigt C, Leitner S, Gahr M (2007). Socially induced brain differentiation in a cooperatively breeding songbird.. Proc R Soc Lond B.

[31] Nottebohm F, Kasparian S, Pandazis C (1981). Brain space for a learned task.. Brain Res.

[32] Catchpole CK, Slater PJB (2008). Bird song..

[33] Mooney R, Hoese W, Nowicki S (2001). Auditory representation of the vocal repertoire in a songbird with multiple song types.. Proc Natl Acad Sci USA.

[34] Nottebohm F, Nottebohm ME, Crane L (1986). Developmental and seasonal changes in the canary song and their relation to changes in anatomy of song-control nuclei.. Behav Neural Biol.

[35] Leitner S, Catchpole CK (2004). Syllable repertoire and the size of the song control system in captive canaries (*Serinus canaria*). J Neurobiol.

[36] Gil D, Naguib M, Riebel K, Rutstein A, Gahr M (2006). Early condition, song learning and the volume of song brain nuclei in the zebra finch (*Taeniopygia guttata*). J Neurobiol.

[37] Brenowitz EA, Lent K, Kroodsma DE (1995). Brain space for learned song in birds develops independently of song learning.. J Neurosci.

[38] DeVoogd TJ, Houtman AM, Falls JB (1995). White-throated sparrow morphs that differ in song production rate also differ in the anatomy of some song-related brain areas.. J Neurobiol.

[39] Jawor JM, Macdougall-Shackleton SA (2008). Seasonal and sex-related variation in song control nuclei in a species with near-monomorphic song, the northern cardinal.. Neurosci Lett.

[40] Hall ZJ, MacDougall-Shackleton SA, Osorio-Beristain M, Murphy TG (2010). Male bias in the song control system despite female bias in song rate in streak-backed orioles *(Icterus pustulatus*).. Brain Behav Evol.

[41] Hamilton KS, King AP, Sengelaub DR, West MJ (1997). A brain of her own: a neural correlate of song assessment in a female songbird.. Neurobiol Learn Mem.

[42] Tobari Y, Nakamura KZ, Okanoya K (2005). Sex differences in the telencephalic song control circuitry in Bengalese finches (*Lonchura striata var. domestica*).. Zoolog Sci.

[43] Washbourne P, Thompson PM, Carta M, Costa ET, Mathews JR (2002). Genetic ablation of the t-SNARE SNAP-25 distinguishes mechanisms of neuroexocytosis.. Nat Neurosci.

[44] Gerber SH, Rah JC, Min SW, Liu X, de Wit H (2008). Conformational switch of syntaxin-1 controls synaptic vesicle fusion.. Science.

