# Supplementary material for: Social Status Affects the Degree of Sex Difference in the Songbird Brain
Source: PLoS One. 2011 Jun 8;6(6):e20723. doi: 10.1371/journal.pone.0020723 (PMC3110770; doi:10.1371/journal.pone.0020723)
Supplement: Table S1 — Measurements of neural properties of male and female white-browed sparrow weavers. (DOC) [file pone.0020723.s001.doc]

Table S1: Measurements of neural properties of male and female white-browed sparrow weavers.

|  |  | **Dominant male** | | **Subordinate male** | | **Dominant female** | |
| --- | --- | --- | --- | --- | --- | --- | --- |
|  |  | **mean** | **s.e.m.** | **mean** | **s.e.m** | **mean** | **s.e.m.** |
| **Gross-morphological parameter** | Total telencephalon volume (mm^3^) | 815.5 | 21.4 | 775.1 | 11.7 | 759.2 | 15.2 |
|  | Total HVC volume (mm^3^) | 3.54 | 0.20 | 2.41 | 0.15 | 1.20 | 0.07 |
|  | HVC cell number (x10^4^ cells) | 42.0 | 3.49 | 29.21 | 2.17 | 16.89 | 1.04 |
|  | HVC cell density (x10^4^ cells/mm^3^) | 24.04 | 1.55 | 24.38 | 1.68 | 29.4 | 0.90 |
|  | Total RA volume (mm^3^) | 1.04 | 0.07 | 0.76 | 0.06 | 0.53 | 0.04 |
|  | RA cell number (x10^4^ cells) | 1.74 | 0.13 | 1.64 | 0.06 | 1.14 | 0.08 |
|  | RA cell density (x10^4^ cells/mm^3^) | 3.34 | 0.14 | 4.12 | 0.24 | 4.29 | 0.22 |
|  | Total area X volume (mm^3^) | 5.04 | 0.60 | 5.35 | 0.64 | 3.67 | 0.20 |
| **mRNA expression level** | Androgen receptor (AR) | 14.38 | 1.01 | 21.18 | 1.81 | 18.21 | 0.74 |
|  | Estrogen receptor α (ER α) | 5.17 | 1.34 | 12.64 | 2.35 | 13.22 | 2.45 |
|  | SNAP-25 | 32.15 | 4.21 | 46.57 | 4.27 | 48.28 | 5.70 |
|  | Synaptoporin (SPO) | 20.59 | 2.92 | 33.02 | 2.38 | 27.36 | 3.85 |
|  | Syntaxin 1B (STX1B) | 18.60 | 2.50 | 28.76 | 1.85 | 31.09 | 2.30 |
